# A methodological comparative analysis of monetary policy and trade openness on economic growth in West Africa: Does language play a role?

**DOI:** 10.1371/journal.pone.0341073

**Published:** 2026-02-05

**Authors:** Seth Acquah Boateng, Bridgette Enimil, Ebenezer Takyi-Danquah

**Affiliations:** 1 School of Economics, University of Cape Coast, Cape Coast, Ghana; 2 School of Business, University of Cape Coast, Cape Coast, Ghana; Instituto Superior de Contabilidade e Administracao de Lisboa - Instituto Politecnico de Lisboa (ISCAL-IPL), PORTUGAL

## Abstract

The study examines the effect of monetary policy and trade openness on economic growth in the ECOWAS countries. Using panel regression analysis and the Random Forest algorithm from the machine learning technique, the study reveals the existence of a major difference between the two language communities. The expansion of the broad money supply has a highly positive effect on economic growth in the two language communities; the impact is much greater in the Francophone countries. But trade openness has contradictory associations; it is positive in the Francophone countries and negative in the Anglophone countries. The Random Forest approach outperforms the standard econometric approach in terms of predictive accuracy (R² = 67.56%), thereby confirming the existence of non-linear associations. The evidence of major differences is linked to the existence of different post-colonial institutions; that is, the common CFA currency institutions in the Francophone countries and the independent national monetary policies in the Anglophone countries. The study implies that ECOWAS countries require distinct policies instead of standard policies.

## 1. Introduction

Policymakers consider economic growth essential in increasing living standards and achieving overall prosperity and world stability [1,2,3]. Important government policies and international trade pacts are critical in shaping the world’s economic growth [4]. Monetary and trade policies significantly contribute to the growth of economies as they influence access to international markets, technology, and resources [5]. Unlike open economies, closed economies respond strongly to changes in expected inflation since the former pay more attention to accurate exchange rate adjustments. Consequently, when the marginal propensity to import is zero, the effect on the budget is absent, but interest rates fall due to the depreciation of the real exchange rate [6]. Free trade may help reduce the adverse effects of contractionary monetary policy on income distribution. A positive trade openness shock decreases both consumption and income inequalities, reducing the rise in inequality caused by the contractionary monetary policy [7]. Adopting a standard monetary policy in a currency zone is associated with less volatility in macroeconomic performance and a less steep Phillips curve. Moreover, it highlights complex interactions between trade openness and monetary policy, implying more inflation and output trade-offs [8].

Colonisation has created distinct institutional frameworks between Anglophone and Francophone countries that shape economic policy effectiveness today. The monetary institutions differ, with Francophone countries sharing the CFA franc, while their Anglophone counterparts maintain their currencies [9,10,11]. Other areas include the legal system, which ranges from common to civil law, and the administrative framework. These institutional legacies of colonialism persist in divergent policy implementation methods, different monetary sovereignty, and differing economic integrations with former colonial masters. Pacquement [12] and Vallin [13]also observe that through the monetary cooperation agreement between France and Africa, France still maintains appreciable economic relations with its erstwhile colonies, while the Anglophone countries mostly follow independent monetary policies. These historical and institutional differences provide a basis on which identical economic policies may turn in different directions across these language groups. These factors flag why it is so important to understand how such colonial legacies shape the effectiveness of macroeconomic policies in ECOWAS [14,15].

Trade openness significantly affects how well monetary policy works in Africa. The research on 37 African countries from 199 to 2015 shows that higher levels of trade openness increase the effectiveness of monetary policy on output growth while reducing its impact on inflation rates. This implies that African monetary authorities must consider the extent of openness to trade when designing monetary policy strategies meant to achieve particular economic goals [16]. Mwinaayelle [17] notes that trade openness significantly affects African countries under fixed exchange rate regimes, suggesting that a stable currency can promote productivity development and economic growth. However, the relationship between monetary policy, primarily through exchange rate arrangements, and trade openness is intricate because floating rates cause instability, which hurts total productivity levels. The recommendation for African nations to adopt fixed rates vis-à-vis the United States dollar is designed to create more certainty in national economies, thus attracting investment while increasing trade openness.

According to Michael et al. [18], there is a strong positive relationship between inflation and broad money growth rates among Mauritius, Nigeria, South Africa, Namibia, and Kenya. Conversely, economic growth is adversely affected by inflation. In these countries, it is evident that an expansion of the money supply leads to increased economic activity. However, an upward trend exists between inflation levels and monetary growth rates. The papers may not mention it explicitly, but it is clear that they are interrelated through exchange rate stabilisation and commercial regulations, respectively, and indirectly influence monetarism, including inflation rate control. An efficient monetary policy always needs to be put in place by governments to control prices that result in a stable economy with improved standards of living.

Colonial legacies left by Britain and France in West Africa embody different strategies for independence and subsequent post-colonial relations, impacting the governance of these countries immensely in their modern economic lives. The British strategy for decolonisation was more about immediate withdrawal, and they gave their colonies complete monetary and fiscal autonomy [9,10,11]. The “clean break” policy allowed for the establishment of independent central banks, the creation of national currencies, and the implementation of autonomous monetary policies in Anglophone countries. In contrast, France followed a more gradual pattern that maintained very significant economic ties through the CFA franc zone. The Franco-African monetary cooperation agreement solidified France’s control over its ex-colonies. The CFA franc was fixed to the French currency, now the euro, with France guaranteeing the free exchange of the currency [19,20].

These varied paths to independence had the consequence of distinct institutional arrangements that persist today. Francophone countries have a monetary union with pooled currency policies, central banking, and foreign exchange reserves managed partly by the French treasury. Anglophone countries had distinct monetary systems with greater policy independence but possibly higher exposure to external shocks. In this study, the classification of languages serves as a proxy for these inherently different institutional frameworks, reflecting not just linguistic variation but also substantial variation in monetary authority, economic integration patterns, and policy implementation regimes that have been inherited from their respective colonial powers [20,19,21,9]. This study answers the following questions (RQ1): How do Monetary Policies and Trade Openness impact Economic growth across ECOWAS countries? (RQ2) Do Monetary Policy and Trade Openness affect Economic Growth differently between francophone and Anglophone countries in West Africa?

The research innovatively combines machine learning techniques with traditional econometrics, uncovering hidden patterns in the impact of colonial institutional frameworks on modern economic policy. In contrast to existing research that has considered ECOWAS as a homogeneous entity, this research breaks new ground by conducting a comparative analysis between Anglophone and Francophone countries to explain how their distinctive colonial heritage affects current policy outcomes. The novelty in the methodology of the study is that it applies Random Forest modelling together with panel regression to create a robust analytical framework for uncovering both linear and non-linear relationships in the environment of policy effectiveness. This two-pronged methodological approach, and its deployment for exploring the heterogeneous effects of monetary policy and trade openness on colonial legacy groups, represents a novel contribution to the literature. Moreover, by explicitly linking institutional differences in history to the performance of contemporary policies, the study offers a new strategy for exploring how West African colonial government structures still affect West African economic development patterns. Not only is the model a contribution to scholarly knowledge, but it also yields useful knowledge for policymakers concerned with regional economic integration and hence is a contribution to theoretical and applied economic research alike.

Aside from the introduction, the remainder of the work is systematised: Section two summarises appropriate literary works on the research topic. Section three provides the theoretical underpinning, data, and assessment technique. Section four presents the empirical findings and discussion. The conclusion, implication, limitation, and future investigation of the study are given in section five.

## 2. Literature review and theoretical framework

This section encapsulates the body of research that has already been done on the subject. More so, we look at earlier studies examining the connections between several essential variables, including the use of money supply, inflation, exchange rate, trade, and the theoretical model.

### 2.1. Monetary policy and economic growth

Broad money, measured as an aspect of monetary policy, is crucial in understanding how to initiate and execute monetary measures. It comprises various financial securities, such as money in circulation and interest-bearing deposits, designed to facilitate transactions and affect the demands of an economy [22]. Studies have shown that monetary policy influences economic performance based on regional and economic contexts. This relationship between money and rate control, on the one hand, and supply into the system, on the other hand, is intricate, being affected by institutional setups, among others [23]. The degree to which economic growth depends on monetary policy varies based on a country’s specific economic conditions and policy implementation decisions. Research has shown that, generally, economic growth rates are positively impacted by money supply increases. In the case of the BRICS bloc, money supply directly affects economic growth, implying that monetary strategies should be made to push up financial inclusion, thus fueling economic growth [24]. However, some discrepancies have emerged regarding the impacts of this instrument, mainly due to knowledge gaps, digital economies, and Islamic monetary policies [25].

Barua et al. [26] discovered that such instruments also have significant implications on general levels of inflation as well as GDP balance in the balance of payments position because they increase private investment through adjusting aggregate demand. Easing inflation and an upsurge in the level of investments are associated with the control of interest rates under normal circumstances. One of the areas where there have been variations in how major monetary policies work is across regions. Monetarily, over the long-term period, monetary policy was more effective in the SAARC nations, while in the short run, private-sector credit was more important for growth [27]. Expansionary monetary policy was more appropriate for achieving economic growth in developing countries [28]. However, it is essential to note that the effectiveness of monetary tools varies across all settings. Factors including economic environment, institutional arrangements, and specific instruments employed significantly affect these outcomes. To get desirable economic growth results, policymakers must design monetary strategies that are in tune with prevailing economic realities in their jurisdictions.

### 2.2. Trade openness and economic growth

Trade boosts economic growth by serving as a stimulus and a development enabler [21]. A country’s economic growth may be influenced by several aspects, including but not limited to trade policies, export composition vis-à-vis importation, and its level of development. Resource allocation within the economy is refined as efficiency in the production process is established through trade, causing an improvement in growth rates. In contrast, it can have different results in different contexts or periods [29]. Moreover, exports and imports do not lead to economic growth in an equal measure but rather stimulate it, as shown by Granger causality tests, which indicate co-integration over the long run. In the short term, however, bi-directional causality exists between commerce and expansion, indicating that each one impacts the other and is mutually influenced [30]. In G20 countries, trade promotes economic growth through more of a supportive role than being its primary driver [31]. Trade positively correlates to growth, but is not a growth engine; therefore, proposed policies cannot increase real per capita income growth [30]. This suggests that trade and FDI, which are otherwise known as foreign direct investment, have been vital components that have seen China experience rapid economic growth by drawing upon globalisation as well as reforming non-market practices contrary to Japan’s more cautious approach during its stages of economic development [32]. Following trade and FDI, China experienced economic development, while Japan changed in response to the global economic order.

Trade openers in Mediterranean countries have been able to experience an increase in economic growth due to trade liberalisation policies, with human capital investment rates acting as growth drivers. However, the slow financial sector has been affected [33]. In summation, commercial and financial openness support the economy’s growth. The interrelated behaviours in a long-term relationship between trade flows and economic growth indicate that the promotion of trade can significantly contribute to the growth of West African countries [34]. This study concludes that cointegration relationships exist between variables, and the long-term relationships between exports, growth, and exchange rates are favourable. International trade is a critical element of economic growth-not the sole factor; other vital factors include domestic policies, financial sector development, and human capital. In addition, the effectiveness of trade as an engine for economic growth may vary depending on the country’s specific economic structure and policy environment. As a result, we should consider combining trade with other enabling factors to achieve sustainable development through this method.

### 2.3. Inflation and economic growth

The link between high inflation and economic development changes in different regions and economic settings; some suggest that it is a negative, others positive or non-linear relationship. The complexity lies in influences from factors such as monetary policy, economic structure, and level of inflation, among others, which vary from country to country. In the ASEAN-5 countries, inflation negatively hinders the growth of the economy in Indonesia and the Philippines, whereas this adverse impact is not felt in Singapore [Su & Soon, 2023]. In Sub-Saharan Africa, Chindengwike [20] points out that inflation has long-term ill effects on growth but correlates positively with short-run growth; hence, it results from both directions. On the other hand, they found a long-run negative relationship between inflation and economic growth and a bidirectional link between economic growth and inflation. High inflation lowers economic development, while low and constant rates can promote it [35]. Also, population growth is a constraint on economic growth and a vital driver of the incidence of poverty [36]. The cointegration analysis and Error Correction Model (ECM) examines the relationship between inflation and economic growth of the GDP and CPI. There is evidence of a long-term relationship between inflation and growth in most countries because both variables are adjusted according to each other in such nations, as found by Ahmmed et al. [35], who investigated the relationship between these issues.

Different countries experience varying sensitivity of inflation to growth. There are variations in the inflation-growth relationship among countries. In some countries, there is a direct relationship between inflation and economic growth, while in others, it is negative. Therefore, in developing countries, several types of inflation can drive growth, with structural and demand-pull inflation being more pronounced than in developed markets, and their inflation thresholds are generally higher for advanced markets [37]. This implies that inflation types other than inflationary finance are more important for growth in developing countries. Although inflation is generally an obstacle to economic development in developing countries, its effects vary depending on the regions or sectors that make up these countries. Policy issues like monetary effectiveness, Foreign Direct Investment, and exchange rate stability play a significant role in the inflation-growth nexus. Therefore, they must consider these things when drawing policies that drive sustainable development.

### 2.4. Exchange rate and economic growth

Multiple studies have shown various direct and indirect causes for the relationship between exchange rates and economic growth. Trade, investment, inflation, and employment are among the things that influence exchange rates in terms of economic growth, even as they may have different implications in different places and conditions. In different environments like Ghana, Nigeria, BRICS countries, and other underdeveloped economies, these studies have indicated that exchange rate movements affect economic growth. For example, Ghana has only one set limit beyond which economic growth is significantly reduced, at 8.97%, which means that exchange rates do not correlate linearly with economic growth [19]. Economic development in Ghana is impaired when exchange rates reach 8.97 percent, and inflation negatively affects low regime growth rates, while it does the opposite for high regimes. The result indicates a weak negative relationship between Nigeria’s exchange rate and GDP growth, while cointegration analysis reveals its long-term significance [38]. For economic growth in Nigeria, it is essential to have a weak negative relationship between the exchange rate and GDP growth and a stable, predictable rate. Tracing the effect of the exchange rate on economic growth in Nigeria through the use of unit root tests that show cointegration and bound tests for long-run relationships between economic factors, Ezebunwo (2024) found that the exchange rate positively affects economic growth in Nigeria such that one unit increase in the exchange rate leads to.3986 units increase in the real GDP in the long run.

High exchange rate volatility negatively influences growth in third-world economies with high financial openness levels and under flexible exchange rate systems, hence the need for policies to bring stability and limit volatility [39]. Volatility in exchange rates in the developing world slows down economic progress; nonetheless, it depends on which exchange rate regime has been adopted and the degree of openness of the financial system. Exchange rates have an asymmetric long-term impact on economic growth in Indonesia that influences trade balance and GDP, implying that for high growth, there should be government intervention to stabilise the IDR/USD exchange rate [40]. Even though some research suggests that fundamental exchange rate undervaluation promotes economic growth, particularly in developing countries, other studies highlight the negative impacts of exchange rate volatility on growth. This demonstrates how complex the exchange rate-growth nexus could imply the need for tailor-made policy interventions to capture gains while averting threats [41].

### 2.5. Application of machine learning models

Machine learning models have increasingly been incorporated into economic research, improving their capacity to forecast, thus offering more profound insights into complex economic phenomena [42]. In contrast with traditional ways of doing models, these models are best suited for identifying non-linear patterns and coping with massive data sets in economics. The use of machine learning techniques crosses various sub-areas of economic study, including forecasting financial markets, macroeconomic analysis, and prediction at a national level [43,44,45]. Furthermore, machine learning models such as LSTM and CNN-BiLSTM have proved effective in predicting business stocks in addition to economic data trends. These models capture nonlinear patterns in dynamic markets, leading to more accurate predictions than the ARIMA method used by most other known models [42]. Macroeconomic forecasters use machine learning models to predict GDP growth rates, which has been demonstrated through studies focusing on China and India [46]. These models, especially during stable economic periods, are generally more accurate than traditional econometric models [46].

The application of Random Forest is found in several fields of economics where it yields versatile and powerful solutions for prediction and classification tasks. Consequently, Random Forest is often preferred when handling large data sets and complex inter-variable behaviours that may be relevant in many economic scenarios [47]. Random Forest Regressor has also been used to forecast enterprise net profit, considering the integration of internal and external factors. This is especially important given the ongoing digital transformation process and the general strategic choice of development path [48]. During the COVID-19 pandemic, alongside Neural Networks, Random Forest was employed to predict economic recession impacts with a high level of accuracy. This also shows why it is essential in crisis management and economic prediction in general [49]. Based on the arguments raised in the paper, a machine learning-based approach is developed and used to identify the effect of monetary policy and trade openness on economic growth in the ECOWAS sub-region.

### 2.6. Theoretical framework

This research was developed based on the endogenous growth theory that economists developed [50,51]. Economic growth is driven by internal elements like knowledge accumulation instead of external elements. Increasing returns driven by technology spillovers are central, according to Romer [51]. There are thus two channels through which a country can be affected in regard to this research. The channels include how money affects economic growth by increasing spending on investment in productive assets and innovation, whereas trade openness contributes to technology transfer and knowledge spillovers [Wen et al., 2023]. There are thus different conditions within Francophone and Anglophone countries. It indicates that the primary cause of economic development is mainly dependent on endogenous factors rather than exogenous contributions. The most important aspects contributing to this cycle include investment in human capital and innovations through government guidance (K et al., 2020). Thus, direct spending on education, training, and technology improves efficiency, leading to self-perpetuating prosperity for nations. In addition, stable prices and encouragement of international trade allow such nations to continue growing within such an environment. Furthermore, it considers increasing returns to scale and technology spillovers where knowledge percolates from one place to another, leading to a total productivity and production increase [Tian and Zhang, 2025]. This clearly defines the research’s scope in terms of the impact of monetary policy and openness to trade on economic growth in ECOWAS, showing how these represent significant aspects of domestic choices that could promote consistent growth prospects in line with the diverse economies of this area.

## 3. Data and methodology

### 3.1. Data description

The analysis uses annual panel data from 15 ECOWAS members over the years 2000–2022, obtained from a variety of reputable sources. Variables used in the study are GDP per capita, broad money, inflation, and trade data. These selected years allow inclusion of significant periods of global economic shock, such as the global financial crisis of 2008 and the global pandemic of COVID-19, to assess if trends relating to monetary policy, trade openness, and economic growth hold regardless of overall conditions in a given time series of data. Including periods of global shock allows a good examination of underlying data as it relates to monetary and trade policies in terms of overall effects. The ECOWAS was chosen because of its composition of both Anglo-speaking and Francophone nations in similar regional settings, as it relates to monetary and trade policies, as a basis of comparison. Definitions of variables used in this analysis appear below in table form in Table 1.

**Table 1:** Variable definition.

Variable	Description and Measurement	Source
GDP per capita	Annual gross domestic product divided by midyear population, measured in current US dollars	WDI
Broad Money	Total volume of monetary assets including narrow money (M1) plus quasi money, expressed as a percentage of GDP	WDI
Inflation Rate	Annual percentage change in consumer price index, reflecting the cost of acquiring a fixed basket of goods and services	WDI
Exchange Rate	Official exchange rate to US dollar, period average	International Financial Statistics (IMF)
Trade Openness	Sum of exports and imports of goods and services as a percentage of GDP	WDI
Language	Dummy variable: 1 for Francophone countries, 0 for Anglophone countries	Author’s compilation based on official languages

World Bank World Development Indicators [WDI, 2024] and International Monetary Fund International Financial Statistics (IMF- IFS, 2024)

### 3.2. Data preprocessing

The raw data for all ECOWAS countries were consolidated into a single dataset for analysis. Missing values, which occurred primarily in economic indicators, were addressed using linear interpolation for time series gaps and mean imputation for random missing observations. Outliers were detected using the standard deviation method (±3σ from the mean) and treated by winsorizing extreme values to maintain data integrity while preserving the overall distribution pattern. All variables were standardised using z-score normalisation, transforming them to have a mean of 0 and a standard deviation of 1, a crucial step for both econometric analysis and machine learning applications in economic research [24,20]. Finally, a thorough quality check was conducted to verify the consistency and integrity of the cleaned and standardised dataset.

### 3.3. Econometric framework

We employed two economic approaches in modelling the relationship between economic growth and key policy variables in the member countries of ECOWAS. We followed a sequential methodology as in Singh and Verma [24] and Useche et al. [52] by starting with baseline OLS models, followed by static panel modelling specifications. The baseline OLS model provides an initial discussion about the relationships between variables, while panel data models are based on unobserved country-specific heterogeneity. The dynamic specification was modelled to include lagged dependent variables as regressors, recognising that the current economic performance would likely be a function of past economic performance.

#### 3.3.1. Baseline OLS model.

The baseline OLS model [Crosby et al., 2010] is specified as:


$$\begin{array}{cc} GDP_{P}erCapita_{i}t= & \;\beta_{\text{0}}+\beta_{\text{1}}BroadMoney_{i}t+\beta_{\text{2}}Inflation_{i}t+\beta_{\text{3}}ExchangeRate_{i}t\\ & +\beta_{\text{4}}Trade_{i}t+\beta_{\text{5}}Language_{i}+\varepsilon_{i}t \end{array}$$ (1)

where i denotes country,  t denotes time period, and $\varepsilon_{i}t$ is the idiosyncratic error term. The model assumes cross-sectional independence and homoscedasticity. Key limitations include potential endogeneity bias and the inability to control for unobserved country-specific effects.

#### 3.3.2. Static panel data model.

The Hausman specification test was used to select between the Fixed Effects (FE) and Random Effects (RE) models (Baltagi, Bresson and Pirotte, 2003). On performing the test, it was found that χ2(4) = 23.26, p < 0.001, which rejects the null hypothesis, suggesting that there is a systematic difference between the FE and RE estimates. This indicates that the country-specific factors are correlated with the explanatory variables. As such, the Fixed Effects model is more appropriate and valid for analysis. The Fixed Effects (FE) model accounts for unobserved country-specific heterogeneity [Fischer, 2010]:


$$\begin{array}{cc} GDP_{P}erCapita_{i}t= & \;\beta_{\text{0}}+\beta_{\text{1}}BroadMoney_{i}t+\beta_{\text{2}}Inflation_{i}t+\beta_{\text{3}}ExchangeRate_{i}t\\ & +\beta_{\text{4}}Trade_{i}t+\alpha_{i}+\varepsilon_{i}t \end{array}$$ (2)

where $\alpha_{i}\;$ captures time-invariant country-specific fixed effects and $\varepsilon_{i}t$ is the idiosyncratic error term.

### 3.4. Machine learning model

#### 3.4.1. Random forest.

The study complemented traditional econometric analysis with a machine learning approach using Random Forest regression to capture potential non-linear relationships and complex interactions. Following recent applications in economic research [53,54], we structured our Random Forest model as follows:


$$\hat{y}=\left(\text{1}/T\right)\sum \left(t=\text{1}toT)h_{t}(x\right)$$ (3)

where $\hat{y}$ is the predicted GDP per capita, T is the number of trees in the forest, and $h_{t}(x)$ represents the prediction from the t−th decision tree.

#### 3.4.2. Training and validation.

We designed a detailed temporal structure for the training and validation framework of our model to preserve the time-series integrity of the data. These involved creating a time-series train-test split spanning the years 2000–2022 and developing a robust out-of-sample predictive capability from it. To illustrate, the above data ranges from 2000 to 2018 and represents almost 80% of the total observations in the training set. This created a substantial training ground where the model could learn from various economic cycles and policy changes over time. The validation set captured data from 2019–2020, representing 10% of the overall dataset, and the test set included the years 2021–2022 (10%): both sets provided us with a chance to see how well our model performs in recent economic conditions. Moreover, five-fold time-series cross-validation was employed while keeping in mind the time structure of all observations because this is highly relevant to economic data, as past events may influence future outcomes.

#### 3.4.3. Feature importance analysis.

We employed multiple complementary approaches to understand the relative impact of different variables on economic growth predictions. First, we used the Mean Decrease in Impurity (MDI) to measure the contribution of each feature to error reduction through all trees in the forest, which is reliable as an indicator of variable importance. Next, we employed permutation importance scores by randomly shuffling the individual feature values and measuring the resulting decrease in the model performance, thus giving another perspective on the importance of a variable. Partial Dependence plots were developed over important variables to exhibit their individual marginal effects on the predicted GDP while controlling for the average effects of all the other variables.

## 4. Empirical result

### 4.1. Descriptive statistics

Table 2 indicates substantial economic differences across ECOWAS nations. It shows that GDP per capita has a large range from $138.71 to $3,928.31, with a mean of $1,033.16, demonstrating considerable economic discrepancies within the area. Broad money, as a proportion of GDP, averages 28.94%, but ranges widely from 5.21% to 113.65%, showing various monetary policy orientations among nations. Inflation rates also indicate a great variety, ranging from −3.50% to 41.51%, with a mean of 6.04%, alluding to various degrees of price stability. The standard deviation exceeding the mean reflects the high inflation variability of the ECOWAS countries. The causes of these inflations range from differences in the monetary structures of the countries due to occasional economic shocks that differentiate economies. Exchange rates average 101.14 with a range from 68.18 to 218.04, indicating differing currency assessments. Trade Openness, evaluated as a percentage of GDP, averages 59.55%, extending from 16.35% to 117.82%, suggesting various degrees of economic openness across ECOWAS states. These figures show the variability in economic situations and policy contexts within the ECOWAS region.

**Table 2:** Descriptive statistics of key variables.

	GDP	Broad Money	Trade	Inflation	Exchange Rate
mean	1033.16	28.94308	59.55187	6.04292	101.1418
std	812.5138	17.5349	19.1247	6.81858	16.15605
min	138.7139	5.21006	16.35219	−3.50259	68.1798
25%	515.4478	18.69012	46.29515	1.304511	94.5278
50%	730.6114	24.82263	57.55883	3.984295	99
75%	1258.964	31.50196	67.95852	9.00	102.9752
max	3928.309	113.6534	117.8167	41.5095	218.037

All variables are standardised. Data covers 15 ECOWAS countries from 2000–2022.

Authors’ calculations based on World Bank WDI (2024) and IMF- IFS (2024)

### 4.2. Time trends of broad money and trade openness

**Fig 1:**
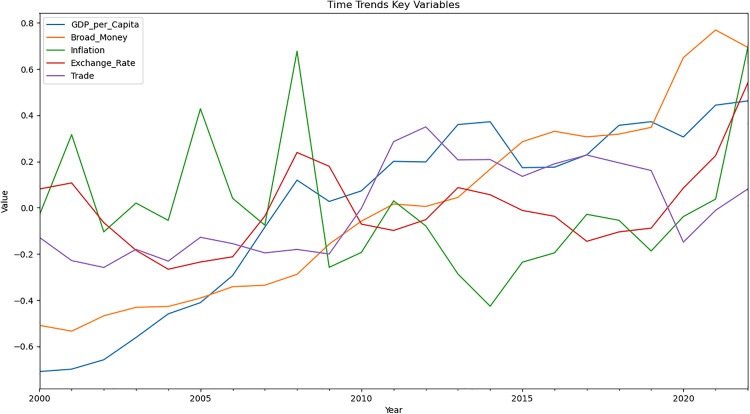
Time trends of the variables.

The line graph presented in Fig 1 shows the trends of key economic variables for ECOWAS countries from 2000 to 2022. GDP per capita and broad money display overall upward trends, indicating economic growth and monetary expansion. Inflation exhibits high volatility, with sharp peaks and troughs throughout the period. The exchange rate remains relatively stable with a slight upward trend, while trade shows fluctuations but maintains a generally consistent level. There’s a noticeable convergence of most variables towards positive values by 2022, suggesting a period of economic stabilisation or growth across the region.

### 4.3. Comparative visualisations between Anglophone and Francophone countries

**Fig 2:**
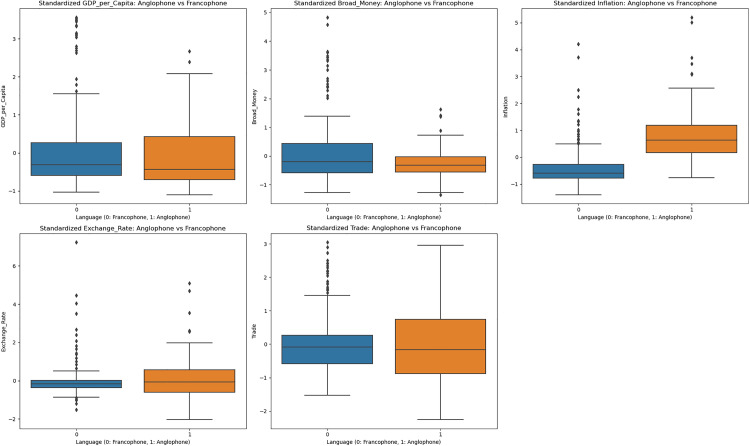
Comparative trends between Anglophone and Francophone countries.

The box plots shown in Fig 2 compare the economic indicators between Anglophone and Francophone countries. GDP per capita shows similar distributions for both groups, with Francophone countries having a slightly higher median. Broad money is more concentrated in Anglophone countries, while Francophone countries show a wider distribution. Inflation is notably higher and more varied in Anglophone countries. Exchange rates are more stable in Francophone countries, likely due to the common currency (CFA franc). Trade openness is similar between the two groups, with Francophone countries showing a slightly higher median and wider distribution.

**Fig 3:**
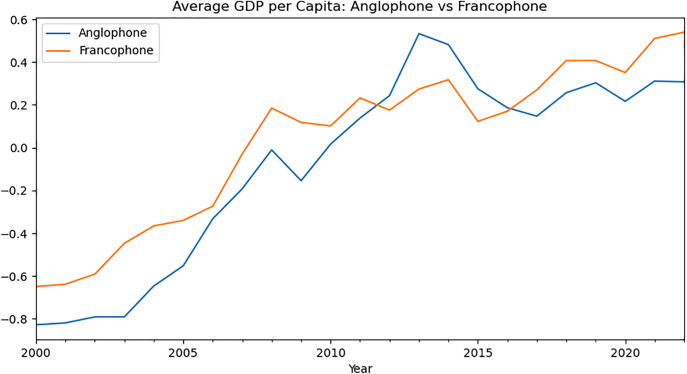
Comparative analysis of GDP of both groups.

Fig 3 compares the GDP per capita between Anglophone and Francophone countries. Both groups show an overall upward trend, indicating economic growth. Francophone countries started with a higher average GDP per capita in 2000 and maintained this lead until around 2010. From 2010 to 2015, Anglophone countries surpassed Francophone ones. After 2015, Francophone countries regained the lead and showed stronger growth, ending the period with a noticeably higher average GDP per capita than Anglophone countries.

### 4.4. Correlation matrix of main variables

**Fig 4:**
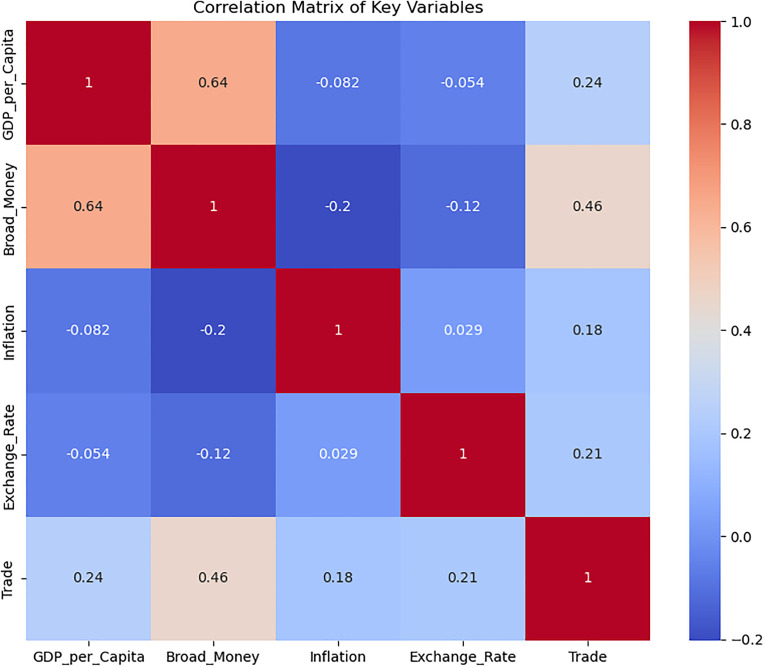
Correlation of the variables.

Fig 4 shows the relationships between the variables. GDP per capita has a strong positive correlation (0.64) with broad money, suggesting that monetary expansion is associated with economic growth. Trade openness has moderate positive correlations with GDP per capita (0.24) and broad money (0.46), indicating that increased trade is linked to economic growth and monetary expansion. Inflation has weak negative correlations with GDP per capita (−0.082) and broad money (−0.2), implying that higher inflation might slightly hinder growth. The exchange rate shows weak correlations with most variables, suggesting limited direct influence on other factors.

### 4.5. Panel unit root test

Table 3 shows the unit root results of the variables. The considered two-panel unit root test: CIPS (Cross-sectional Im-Pesaran-Shin) and CADF (Cross-sectional Augmented Dickey-Fuller). The test result showed that all the variables became stationary at the first difference.

**Table 3:** Panel unit root result.

Variable	At Level				First Difference			
	CIPS_stat	CIPS_p	CADF_stat	CADF_p	CIPS_stat	CIPS_p	CADF_stat	CADF_p
**GDP per capita**	−2.105086	0.035	−2.1050867	0.035	−4.469987	0.000	−4.469987	0.000
**Broad Money**	−2.021017	0.043	−2.02101771	0.043	−5.585843	0.000	−5.585843	0.000
**Trade Openness**	−2.387286	0.016	−2.3872869	0.016	−4.869031	0.000	−4.869031	0.000
**Exchange Rate**	−3.444399	0.000	−3.4443995	0.000	−5.510104	0.000	−5.510104	0.000
**Inflation**	−3.625563	0.000	−3.6255638	0.000	−5.322907	0.000	−5.322907	0.000

Authors’ analysis using data from World Bank WDI (2024)

### 4.6. Regression results

The OLS model was conducted as a baseline model for comparison. The result shown in Table 4 reveals that broad money has a strong positive association with GDP per capita, significant at the 1% level. It shows that a one standard deviation increase in broad money is associated with a 72% standard deviation increase in GDP per capita, holding other factors constant. This suggests that monetary expansion has a substantial positive impact on economic growth in the region. Trade openness, however, shows a significant negative relationship with GDP per capita at the 5% level. A one-standard-deviation increase in trade openness is associated with a 10.7% standard deviation decrease in GDP per capita, ceteris paribus. Inflation and exchange rates show positive but statistically insignificant relationships with GDP per capita. The model’s R-squared value of 0.429 indicates that it explains about 42.9% of the variation in GDP per capita, which is moderate for cross-sectional data. The F-test is significant at the 1% level, confirming the statistical significance of the model. The Variance Inflation Factors (VIF), as seen in Table 5 for all variables, are below 2, indicating no serious multicollinearity issues in the model.

**Table 4:** Linear regression.

GDP Per Capita	Coef.	St.Err.	t-value	p-value	[95% Conf	Interval]	Sig
Broad Money	0.720	.051	14.22	0.000	0.621	0.820	***
Trade Openness	−0.107	.052	−2.070	0.040	−0.208	−0.005	**
Language: base 0		.	.	.	.	.	
1	0.123	0.107	3.150	0.005	0.088	0.333	***
Inflation	0.049	.053	3.920	0.002	0.056	0.154	***
Exchange Rate	0.048	.044	3.090	0.005	0.038	0.133	***
Constant	−0.041	0.054	−0.750	0.452	−0.148	0.066	
Mean dependent var		−0.000	SD dependent var			1.000	
R-squared		0.429	Number of obs			345	
F-test		50.881	Prob > F			0.000	
Akaike crit. (AIC)		796.908	Bayesian crit. (BIC)			819.969	

*** p < .01, ** p < .05, * p < .1

*Authors’ analysis using data from World Bank WDI (2024)*

**Table 5:** Variance inflation factor.

	VIF	1/VIF
Broad Money	1.521	0.657
Inflation	1.684	0.594
Exchange rate	1.127	0.888
Trade Openness	1.58	0.633
1. language	1.509	0.663
**Mean VIF**	**1.484**	.

Authors’ analysis using data from World Bank WDI (2024)

### 4.7. Outcome of the fixed effect model

The fixed effects model was conducted to check for country-specific fixed effects, controlling for time-invariant characteristics of each country. As seen in Table 6, broad money maintains a strong positive association, with a one standard deviation increase corresponding to a 54.6% standard deviation increase in GDP per capita, significant at the 1% level. Inflation shows a negative relationship, with a one standard deviation increase leading to an 8.4% standard deviation decrease in GDP per capita, significant at the 5% level. The exchange rate demonstrates a positive relationship, where a one standard deviation increase is associated with a 13% standard deviation increase in GDP per capita, significant at the 1% level. Interestingly, trade openness maintains a significant negative relationship, with a one standard deviation increase corresponding to a 21.1% standard deviation decrease in GDP per capita, significant at the 1% level. This model accounts for country-specific fixed effects, controlling for time-invariant characteristics of each country, and explains 28.9% of the within-country variation in GDP per capita.

**Table 6:** Fixed effects test result.

GDP Per Capita	Coef.	St.Err.	t-value	p-value	[95% Conf	Interval]	Sig
Broad Money	0.546	0.052	10.43	0.000	0.443	0.649	***
Inflation	−0.084	0.038	−2.21	0.028	−0.159	−0.009	**
Exchange Rate	0.130	0.030	4.25	0.000	0.070	0.189	***
trade	−0.211	0.045	−4.71	0.000	−0.299	−0.123	***
Constant	0.00	0.024	0.00	1.000	−0.048	0.048	
Mean dependent var		−0.000	SD dependent var			1.000	
R-squared		0.289	Number of obs			345	
F-test		33.096	Prob > F			0.000	
Akaike crit. (AIC)		420.850	Bayesian crit. (BIC)			440.068	

*** p < .01, ** p < .05, * p < .1

Authors’ analysis using data from World Bank WDI (2024)

### 4.8. Comparative analysis: Anglophone vs Francophone countries

Table 7 reveals significant differences in the effects of monetary policy and trade openness between Anglophone and Francophone ECOWAS countries. Broad Money shows a strong positive relationship with GDP per capita, with a coefficient of 0.6407 (significant at 1% level), indicating that a one standard deviation increase in Broad Money is associated with a 64.07% standard deviation increase in GDP per capita. However, the interaction term Broad_Money_anglo (−0.4093, significant at 1% level) suggests that this effect is substantially weaker in Anglophone countries. Similarly, while Trade shows a positive but marginally significant effect (0.1290, significant at the 10% level) on GDP per capita overall, the interaction term Trade_anglo (−0.4477, significant at 1% level) indicates a significantly negative effect of trade openness on GDP per capita in Anglophone countries. Inflation and the Exchange Rate do not show statistically significant effects. The model explains 47.6% of the variance in GDP per capita (Adjusted R-squared: 0.476), and the F-statistic (45.61) indicates that the model is statistically significant overall.

**Table 7:** Regression result with language effect.

Variable	Coefficient	Std. Error	t-statistic	P-value
Broad_Money_franco	0.6407	0.060	10.691	0.000***
Broad_Money_anglo	−0.4093	0.141	−2.907	0.004***
Trade_franco	0.1290	0.075	1.715	0.057*
Trade_anglo	−0.4477	0.094	−4.777	0.000***
Inflation	0.0448	0.051	0.875	0.382
Exchange_Rate	−0.0158	0.043	−0.366	0.714
Intercept	−0.0364	0.052	−0.705	0.481
Model Statistic				
Adj. R-squared	0.476			
F-statistic	45.61			

*** p < .01, ** p < .05, * p < .1. Franco coefficients represent base effects for Francophone countries. Anglo coefficients represent interaction effects (additional impact) for Anglophone countries. Total effect for Anglophone countries = Franco coefficient + Anglo coefficient.

Authors’ analysis using data from World Bank WDI (2024)

### 4.9. Machine learning approach

Random Forest model was adopted as a machine learning approach to complement the traditional econometric analysis. The model, as seen in demonstrates strong predictive power for GDP per capita in ECOWAS countries. With an R-squared value of 67.56%, this model explains a substantially higher proportion of the variance in GDP per capita compared to the OLS (42.9%) and fixed effects (28.9%) models. This improved performance suggests that the Random Forest captures complex, non-linear relationships between the predictors and GDP per capita that the linear models may have missed. The Mean Squared Error of 0.4027 indicates a moderate level of prediction accuracy.

#### 4.9.1. Feature importance analysis.

**Fig 5:**
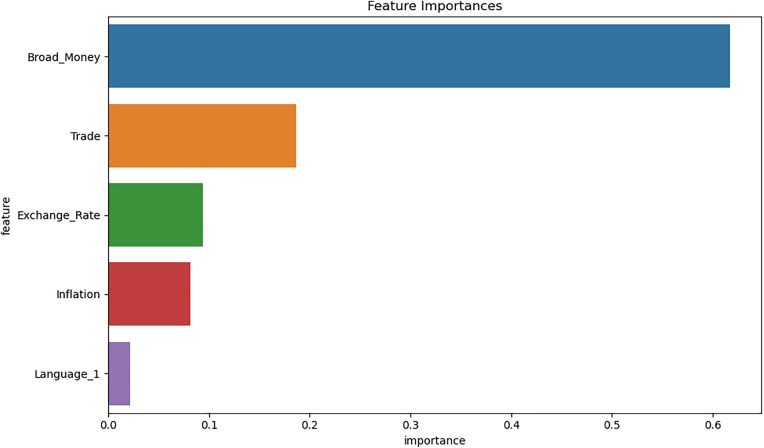
Feature importance analysis.

Fig 5 displays the relative importance of different features in the Random Forest model for predicting GDP per capita. Broad Money emerges as the most crucial feature, with an importance score of 0.67, higher than all other variables. This aligns with the strong positive relationship observed in the regression models. Trade is the second most important feature with a score of 0.22, followed by Exchange Rate and Inflation, 0.12 and 0.0.03 respectively. Language has the least importance in the model. This ranking provides insights into which factors the Random Forest model considers most influential in determining GDP per capita.

**Fig 6:**
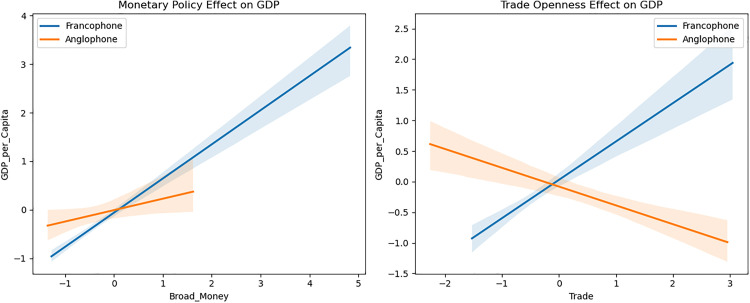
Monetary policy and trade openness among both groups.

Fig 6 shows distinct differences in the effects of monetary policy and trade openness between Francophone and Anglophone countries. The effect of monetary policy on GDP per capita is strongly positive for Francophone countries, as indicated by the steep upward slope of the blue line. In contrast, the effect is much flatter for Anglophone countries, consistent with the negative interaction term in the regression. The right panel demonstrates an increased trade openness is associated with higher GDP per capita in francophone countries. However, for Anglophone countries, the relationship is negative, aligning with the interaction term in the regression.

### 4.10. Robustness checks

The Robustness result, as presented in Table 8, shows the stability and consistency of our findings across different model specifications and periods. The strong positive relationship between Broad Money and GDP per capita remains significant across all specifications, confirming its robust impact on economic growth. However, the magnitude of this effect varies, being strongest in the post-crisis period (0.7356) and weakest with 3 lags (0.4373). Trade openness shows inconsistent effects, being insignificant in most specifications but positive and marginally significant with 3 lags. Inflation’s impact varies across specifications, being significantly negative with 3 lags but positive in the post-crisis period. The exchange rate remains largely insignificant across all models. The pre-and post-crisis comparisons reveal structural changes, with the intercept shifting from significantly negative pre-crisis to positive post-crisis. The log-log model maintains the significance of Broad Money, suggesting the relationship holds even under different functional forms. These checks reinforce the importance of monetary policy while highlighting the sensitivity of other variables to model specifications and periods.

**Table 8:** Robustness checks – alternative model specifications.

Variable	1 Lag	2 Lags	3 Lags	Pre-crisis	Post-crisis	Log-log
Intercept	0.0042 (0.043)	0.0077 (0.045)	0.0103 (0.047)	−0.2993*** (0.049)	0.1011* (0.058)	0.3009 (0.308)
Broad Money	0.6335*** (0.052)	0.5334*** (0.054)	0.4373*** (0.056)	0.4480*** (0.083)	0.7356*** (0.066)	0.4737*** (0.141)
Trade	−0.0511** (0.054)	0.0303*** (0.056)	0.0999** (0.057)	−0.0280 *** (0.077)	−0.0685** (0.067)	0.0487*** (0.166)
Inflation	0.0044 ** (0.045)	−0.0610*** (0.046)	−0.0831* (0.048)	−0.0714 (0.047)	0.1849*** (0.067)	0.3254 (0.356)
Exchange Rate	0.0319** (0.047)	−0.0083 (0.049)	−0.0338** (0.050)	−0.0717 (0.049)	0.0396 *** (0.060)	0.2403*** (0.725)

Authors’ analysis using data from World Bank WDI (2024)

## 5. Discussion

This study investigated the different effects of monetary policy and trade openness on economic growth between Anglophone and Francophone countries in the ECOWAS region, employing traditional econometric and modern machine learning approaches. According to the empirical results, broad money has a strong positive relationship with economic growth in ECOWAS countries, where a standard deviation increase in broad money generates a 72% increase in GDP per capita. Such a finding is consistent with Singh and Verma’s [24] study on BRICS countries, wherein expansion of monetary supply has also been found to significantly contribute to economic growth. Likewise, our results reinforce the conclusions of Usmabn et al.’s [28] study regarding the effectiveness of expansionary monetary policy in developing economies. However, the current research indicates that the effect magnitude is considerably greater than that of previous research, implying that ECOWAS countries might be quite sensitive to monetary policy interventions, probably because of their developing financial markets and monetary frameworks. The results regarding trade openness are quite complicated and differ among different countries across the region; Anglophone countries display a distinct pattern from that of Francophone ones. The negative relationship between trade openness and per capita GDP in Anglophone countries is in contrast with findings by Abendin & Duan [21], which showed that trade had generally positive effects in Africa.

However, the result is more in consonance with Awadzie et al.‘s [19] findings in Ghana, which discovered threshold effects on the relationship between trade and growth. Intriguingly, the Francophone countries showed a much more positive trade effect, which is in line with findings by Rumbia et al. [40] regarding asymmetric international trade impacts. Hence, there might be differences in colonial histories and institutional frameworks, leading to trade policy effectiveness being influenced greatly by historical and institutional parameters in the ECOWAS region. It is noted from the analysis that there exist differences in policy effectiveness in monetary terms for different countries of ECOWAS as far as languages are concerned. Francophone countries show relatively stronger positive responses to monetary interventions. The findings thus extend the work of Ekpo & Effiong [16] on monetary policy effectiveness in Africa by detailing the importance of colonial legacy in policy transmission. While findings by Chindengwike [20] produced results that pooled all of sub-Saharan Africa, evidence by us showed considerable differences according to language groupings. These distinctions were again proven by the Random Forest model, where language group emerged as a strong determinant of the effects of policy, thereby adding knowledge to understand regional economic dynamics as not been captured before using other studies.

## 6. Conclusion

The study analysed the differential effect of monetary policy and trade openness on economic growth in Anglophone and Francophone ECOWAS countries from the year 2000–2022. The findings discovered that there is heterogeneity in policy effectiveness across language groups, and the Francophone countries respond more positively to monetary interventions. Broad money had a strong positive relationship with economic growth, while trade openness effects varied substantially between the two language groups. Several policy recommendations were deduced from these findings. The first is that monetary authorities in ECOWAS should begin to consider differentiated monetary policies for awareness of dissimilar responsiveness to Anglophone and Francophone economies. Second, tailor-made trade policies should centre on those issues as a result of Anglophone countries where attention has not provided favourable impacts. Thirdly, the focus should be put on harmonising monetary and trade policies as part of the regional integration initiative while respecting the unique institutional frameworks of both language groups. Future research may assess the particular transmission mechanisms underlying these differences or assess how digital financial innovation could change policy effectiveness within these groups. Strengthening institutional coordination between the Anglophone and Francophone central banks may, likewise, enhance policy effectiveness across the region. Despite the study’s contribution, this study is not devoid of limitations that point toward some potential avenues for future research [55,56]. The study overlooks informal trade, which is extremely significant in West Africa. Further studies will have to examine specific transmission channels propelling Anglophone and Francophone economies apart, witness how digital financial innovation influences policy effectiveness among these groups, and look into the role of informal economic activities. Scholars can also investigate how enhancing institutional coordination between Anglophone and Francophone central banks can boost regional policy effectiveness.

## Supporting information

S1 Data Research Dataset. (CSV)
